# A Phage Cocktail To Control Surface Colonization by Proteus mirabilis in Catheter-Associated Urinary Tract Infections

**DOI:** 10.1128/spectrum.02092-22

**Published:** 2022-10-04

**Authors:** Arezoo Mirzaei, Jeroen Wagemans, Bahram Nasr Esfahani, Rob Lavigne, Sharareh Moghim

**Affiliations:** a Department of Bacteriology and Virology, School of Medicine, Isfahan University of Medical Sciences, Isfahan, Iran; b Department of Biosystems, KU Leuven, Leuven, Belgium; Institut Pasteur

**Keywords:** phage cocktail therapy, *Proteus mirabilis*, catheter-associated urinary tract infections (CAUTIs), biofilm, quorum sensing, phantom bladder model, catheter-associated urinary tract infections, biofilms

## Abstract

Proteus mirabilis is a biofilm-forming bacterium and one of the most common causes of catheter-associated urinary tract infections (CAUTIs). The rapid spread of multidrug-resistant P. mirabilis represents a severe threat to management of nosocomial infections. This study aimed to isolate a potent phage cocktail and assess its potential to control urinary tract infections caused by biofilm-forming P. mirabilis. Two lytic phages, Isf-Pm1 and Isf-Pm2, were isolated and characterized by proteome analysis, transmission electron microscopy, and whole-genome sequencing. The host range and effect of the phage cocktail to reduce the biofilm formation were assessed by a cell adhesion assay in Vero cells and a phantom bladder model. The samples treated with the phage cocktail showed a significant reduction (65%) in the biofilm mass. Anti-quorum sensing and quantitative real-time PCR assays were also used to assess the amounts of transcription of genes involved in quorum sensing and biofilm formation. Furthermore, the phage-treated samples showed a downregulation of genes involved in the biofilm formation. In conclusion, these results highlight the efficacy of two isolated phages to control the biofilms produced by P. mirabilis CAUTIs.

**IMPORTANCE** The rapid spread of multidrug-resistant (MDR) and extensively drug-resistant (XDR) bacterial strains and biofilm formation of bacteria have severely restricted the use of antibiotics and become a challenging issue in hospitals. Therefore, there is a necessity for alternative or complementary treatment measures, such as the use of virulent bacteriophages (phages), as effective therapeutic strategies.

## INTRODUCTION

Proteus mirabilis is one of the most common causes of catheter-associated urinary tract infections (CAUTIs) (1). This opportunistic bacterium develops biofilm on the surface of a newly installed urinary catheter that is antibiotic resistant and difficult to remove. As one of the most important virulence factors of P. mirabilis, fimbriae mediate binding to host cells and catheters, which are thus involved in forming the dense biofilms on catheter surfaces, contributing to its virulence (2). Moreover, this bacterium produces urease, accountable for urea hydrolysis to carbon dioxide and ammonia, increasing the urine pH to more than 8.3 and thus leading to precipitation of calcium and magnesium ions and the formation of urinary stones composed of magnesium ammonium phosphate (struvite) and calcium phosphate (apatite) (3). The catheterization length may be an essential risk of CAUTIs; most long-term-catheterized (>28 days) patients develop CAUTIs, while only 10 to 50% of short-term-catheterized (<7 days) patients may develop the associated infections (4).

In recent years, the rapid spread of multidrug-resistant (MDR) and extensively drug-resistant (XDR) bacterial strains has severely restricted use of antibiotics. Furthermore, the rapid spread of MDR P. mirabilis represents a severe threat to management of nosocomial infections (5). This makes treating CAUTIs complicated, accentuating the necessity for alternative or complementary treatment measures, such as using virulent bacteriophages (phages) as therapeutic strategies (6). Phages are potential antibacterial agents because they self-replicate inside host cells and are eliminated from the body in their absence (7). Phages have some advantages that make them appropriate antimicrobial agents, including the ability to multiply at the site of infection and target only specific bacteria with no effect on commensal flora, activity against biofilms and other surface structures that contribute to antibiotic resistance, and susceptibility to genetic modification (8). However, the emergence of bacterial resistance to the phage and the chance of undesirable adaptational outcomes of phage-bacterium interaction have been reported (9). Also, sometimes phages would induce the phage-immune system and refuse other phage infections (10). This could be circumvented by combining many phages, so-called phage cocktails, in a single preparation (11). Phage cocktails can increase the range of antimicrobial activity against multiple strains of the target bacterial species (8). Catheters coated with a phage cocktail composed of P. mirabilis phages showed a significant reduction of P. mirabilis biofilm formation in a dynamic biofilm model simulating CAUTIs (12). Biofilm eradication has become challenging in hospitals. Various phage proteins, such as preneck appendage or lytic protein, were shown to prevent or remove previously formed biofilms by breaking down the extracellular matrix or killing bacteria in the biofilm structure of staphylococcal species (13, 14). In Iran, similar to other countries, the prevalence of MDR bacteria is increasing. Therefore, alternative strategies to control MDR bacterial infections, including phages and the combination of phages (phage cocktails), have received more attention among researchers. We aimed to isolate and characterize two lytic phages which infected P. mirabilis associated with CAUTIs. We examined host range, biological properties, complete genome sequencing, and their efficacy in controlling biofilm formation using an *in vitro* biofilm model.

## RESULTS

### Phage morphology and host range.

**FIG 1:**
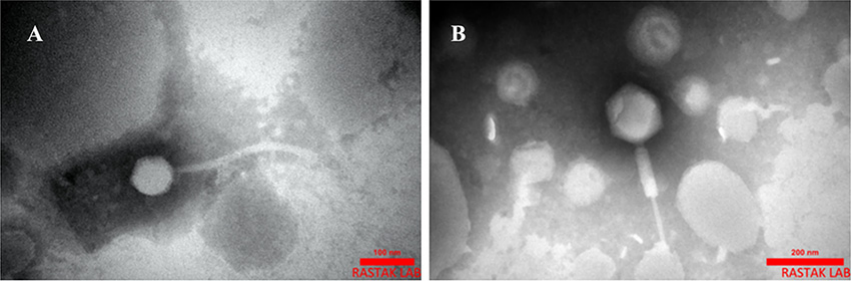
Transmission electron micrograph of the isolated phages. (A) Isf-Pm1; (B) Isf-Pm2. Scale bars indicate 100 and 200 nm, respectively.

Lytic phages were isolated from municipal sewage using P. mirabilis strain ATCC 7002 as the host. Based on the initial screening of the plaque morphology, two different phages, Isf-Pm1 and Isf-Pm2, were isolated and plaque purified. Transmission electron microscopy (TEM) revealed that phage Isf-Pm1 has a siphovirus morphology with an icosahedral head (166.6 by 137.03 nm) and a tail (about 277.7 nm) (Fig. 1A). Phage Isf-Pm2 has an icosahedral head (86.64 by 75.67 nm) with a tail (270.2 nm long) that resembles the myovirus morphology (Fig. 1B). On semisolid nutrient agar, bacterial lawn Isf-Pm1 and Isf-Pm2 produce clear plaques with sizes of up to 1 and 7 mm in diameter, respectively, after incubation at 37°C (see Fig. S1 at https://www.researchgate.net/publication/363250612_Supplementary_Figures_and-T00les).

Stability testing of the phage cocktail (Isf-Pm1 and Isf-Pm2) against different pHs highlighted that the phages were stable at pH ranges from 3 to 13, with a slight decrease in lysis halo at pH 3 and an optimum at pH 7 (Fig. S2). The lysis capacity of phages was assessed at different temperatures ranging from 4°C to 70°C. However, the optimal temperature for lytic activity was 37°C, and the infectivity was reduced at 70°C. The activity of the virus was not affected by chloroform treatment (Fig. S3). Among 40 P. mirabilis isolates, 33 were MDR as determined by antibiotic resistance profiles (Fig. S4). To investigate the host specificity of both phages, all 40 clinical P. mirabilis isolates were used. The results showed that Isf-Pm1 could lyse 30/40 (75%) of the clinical isolates, whereas Isf-Pm2 could lyse 34/40 (85%) of the P. mirabilis isolates. Thirty isolates overlapped for the phages (Table S1). The phage cocktail (1:1; 10^8^ PFU of each phage) was able to affect 85% of all strains. Neither phage showed lytic activity against *Enterobacteriaceae* (Escherichia coli [ATCC 25922]), Klebsiella pneumoniae (ATCC 700603), Shigella sonnei (ATCC 9290), or Salmonella enterica serovar Typhi (ATCC 6539).

### Biological characterization of the phages.

**FIG 2:**
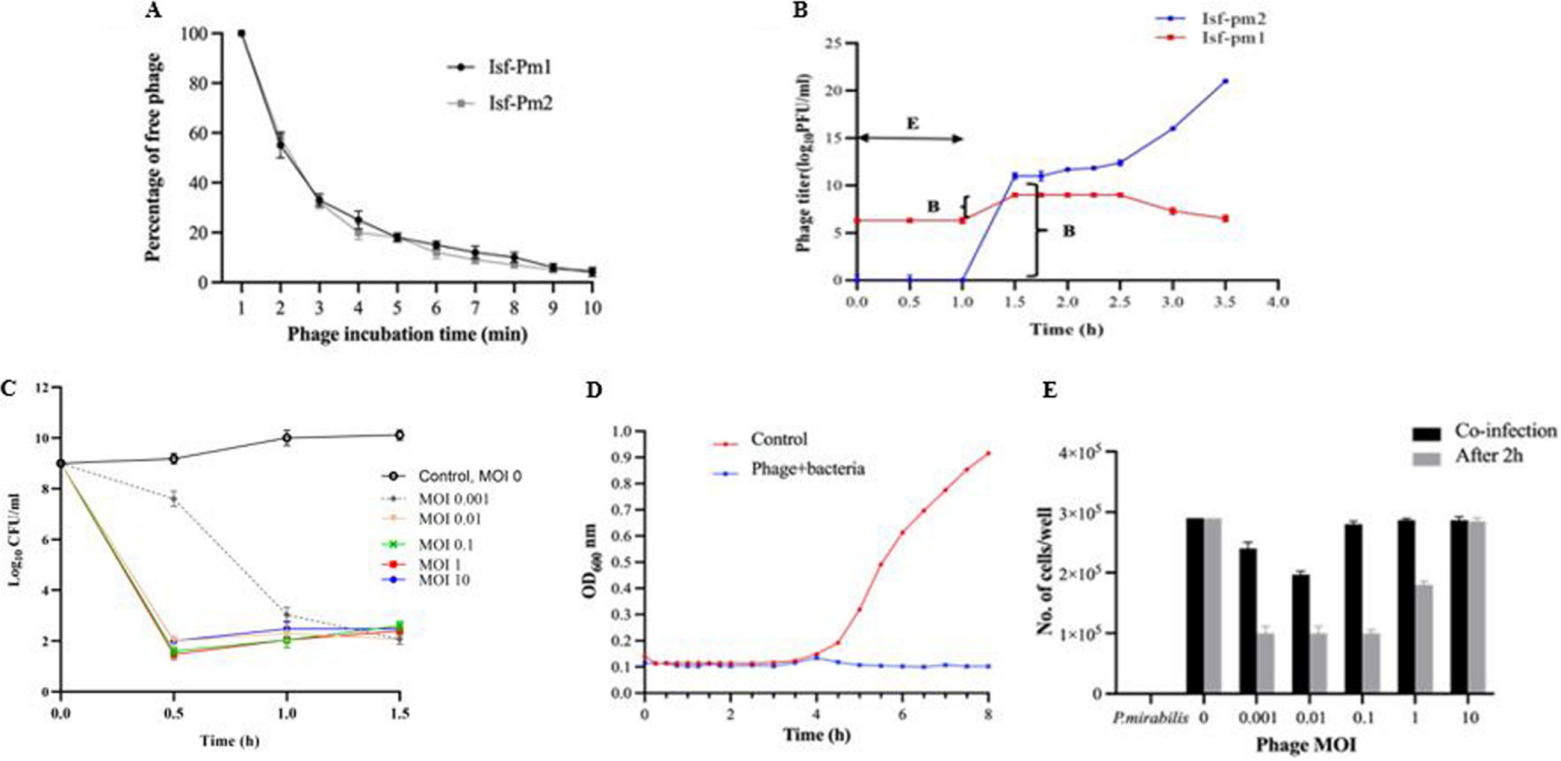
Biological properties of phages Isf-Pm1 and Isf-Pm2. (A) Adsorption curve of Isf-Pm1 and Isf-Pm2. (B) One-step growth curve of the phages to host bacterial strain. E, eclipse; B, burst size. (C) Optimal phage cocktail at different MOIs against host bacterial strain. P. mirabilis ATCC 7002 (10^8^ CFU/mL) was infected with the phage cocktail at different MOIs and cultured for 1.5 h. The control experiment was performed using equal volumes of nutrient broth and bacteria. (D) Bacterial reduction assay for phage cocktail. The OD_600_ of infected and noninfected P. mirabilis (10^8^ CFU/mL) with a phage cocktail (10^8^ PFU of each phage; 1:1) was determined every 20 min for 8 h. (E) Cell survival assay of phage cocktail on Vero cells. Vero cells were seeded in a 96-well plate (0.5 × 10^4^ cells/well) and were infected with 10^6^ CFU of host bacterial strain. Phage cocktail at different MOIs (0.001, 0.01, 0.1, and 1) was added to the wells at the same time or 2 h after infection with the bacteria. Phage with MOI 0 considered as the cell control; Vero cell, Phage control: phage with MOI 10 without bacteria, MOI 1, 0.1, and 0.01 of phage with exposure of P. mirabilis. The difference between phage treatment at coinfection and postinfection was statically significant for phage MOIs of 0.001 0.01, and 1 (*P* < 0.0001). Data were analyzed by two-way ANOVA with *post hoc* test analysis by comparing each treatment group to the other for statistical significance. All experiments were performed independently in triplicate with triplicate assay, and the results are shown as means ± SD.

The absorption rate of phage Isf-Pm1and Isf-Pm2 onto P. mirabilis was investigated, and the results showed that approximately 70% of the phage particles were adsorbed within 3 min, and the level of adsorption was nearly 100% at 10 min postinfection, with an adsorption rate constant (*k*) of 3.49 × 10^−8 ^mL/min (Fig. 2A). A one-step growth experiment showed a latent period of 60 min for both Isf-Pm1 and Isf-Pm2, followed by the lysis phase, which lasted for 3.5 h. The burst sizes were estimated 46 and 666 phage particles per infected bacterium for Isf-Pm1 and Isf-Pm2, respectively (Fig. 2B). To determine the optimal multiplicity of infection (MOI) of the phage cocktail, the phage cocktail was added to a P. mirabilis ATCC 7002 (10^8^ CFU/mL) culture at different MOIs. As shown in Fig. 2C, a reduction in phage cocktail-infected P. mirabilis was observed at different MOIs. After 30 min of treatment, reductions of 84% and 82% were seen at MOIs of 1 and 0.1, respectively. At a higher MOI (i.e., 10), the bacterial titer decreased 78% in 30 min.

We also monitored the growth rate of P. mirabilis in the presence of the phage cocktail for 8 h. As shown in Fig. 2D, the phage cocktail could reduce the growth of P. mirabilis significantly compared to the control. After 8 h, no emergence of phage resistance was observed.

### Cell survival assay.

The safety of the phage cocktail was examined in the Vero cell line in the presence of P. mirabilis ATCC 7002. The phage cocktail was added at different MOIs (0.01, 0.1, and 1) to the cells in the presence of 10^8^ CFU/mL of P. mirabilis. The viability of the cells decreased in conjunction with decreasing MOIs, except for an MOI of 0.001, at which cell viability was slightly higher than at an MOI of 0.01. Vero cells that were treated only with bacteria were all disrupted. The survival rate of the cells was decreased when the infected cells were treated with the phage cocktail 2 h postinfection. The viability of Vero cells was higher at an MOI of 1 than at other MOIs (Fig. 2E). The 3-(4,5-dimethyl-2-thiazolyl)-2,5-diphenyl-2H-tetrazolium bromide (MTT) assay confirmed the safety of the phage cocktail in the Vero cell line (Fig. S5).

### Genome analysis.

Phage Isf-Pm1 has a linear double-stranded DNA with the size of 58,354 bp and a GC content of 46.95%. BLASTn searches revealed that Isf-Pm1 is closely related to Proteus phage pPM-01 (99% coverage and 96.98% sequence identity; GenBank accession no. NC_028812) (15). A Virus Intergenomic Distance Calculator (VIRIDIC) intergenomic distance analysis confirmed that Isf-Pm1 belongs to the pPM-01 species, which is a yet-unclassified phage of the proposed “*Pimunavirus*” genus (Fig. S6).

Proteus phage Isf-Pm2 has a linear double-stranded DNA with 167,727 bp and a 35.43% GC content. The presence of 11 tRNA genes was observed in this phage genome. BLASTn analysis showed that Isf-Pm2 is closely related to Escherichia phage D5505 (98% coverage and 97.8% sequence identity). An intergenomic distance analysis confirmed that Isf-Pm2 is an isolate from the same species as D5505, belonging to the *Tequatrovirus* genus of the *Tevenvirinae* subfamily in the *Straboviridae* family (Table 1 and Fig. S7).

**TABLE 1:** Characteristics of isolated phages

Phage	GenBank accession no.	GC content (%)	Genome size (bp)	Morphology
Isf-Pm1	OL741431	46.95	58,354	Siphovirus
Isf-Pm2	OL741432	35.43	167,727	Myovirus

Genome analysis revealed that both phages are virulent and do not harbor any lysogeny-associated genes. Furthermore, no known toxins or virulence-associated or antibiotic resistance proteins were detected *in silico*, making them potentially suitable for therapeutic purposes.

Structural protein analysis of Isf-Pm1 and Isf-Pm2 by SDS-PAGE under denaturing conditions revealed nine structural protein bands (Fig. S8A), with molecular weights ranging from 10 to 180 kDa. The clear band at 10 kDa in a nondenaturing gel with P. mirabilis ATCC 7002 overlays was supposed to be related to endolysins (Fig. S8B), although the matrix-assisted laser desorption ionization–time of flight mass spectrometry (MALDI-TOF) mass spectrometry needs confirmation.

### Efficacy of the phage cocktail against biofilm, quorum sensing (QS), and adhesion.

**FIG 3:**
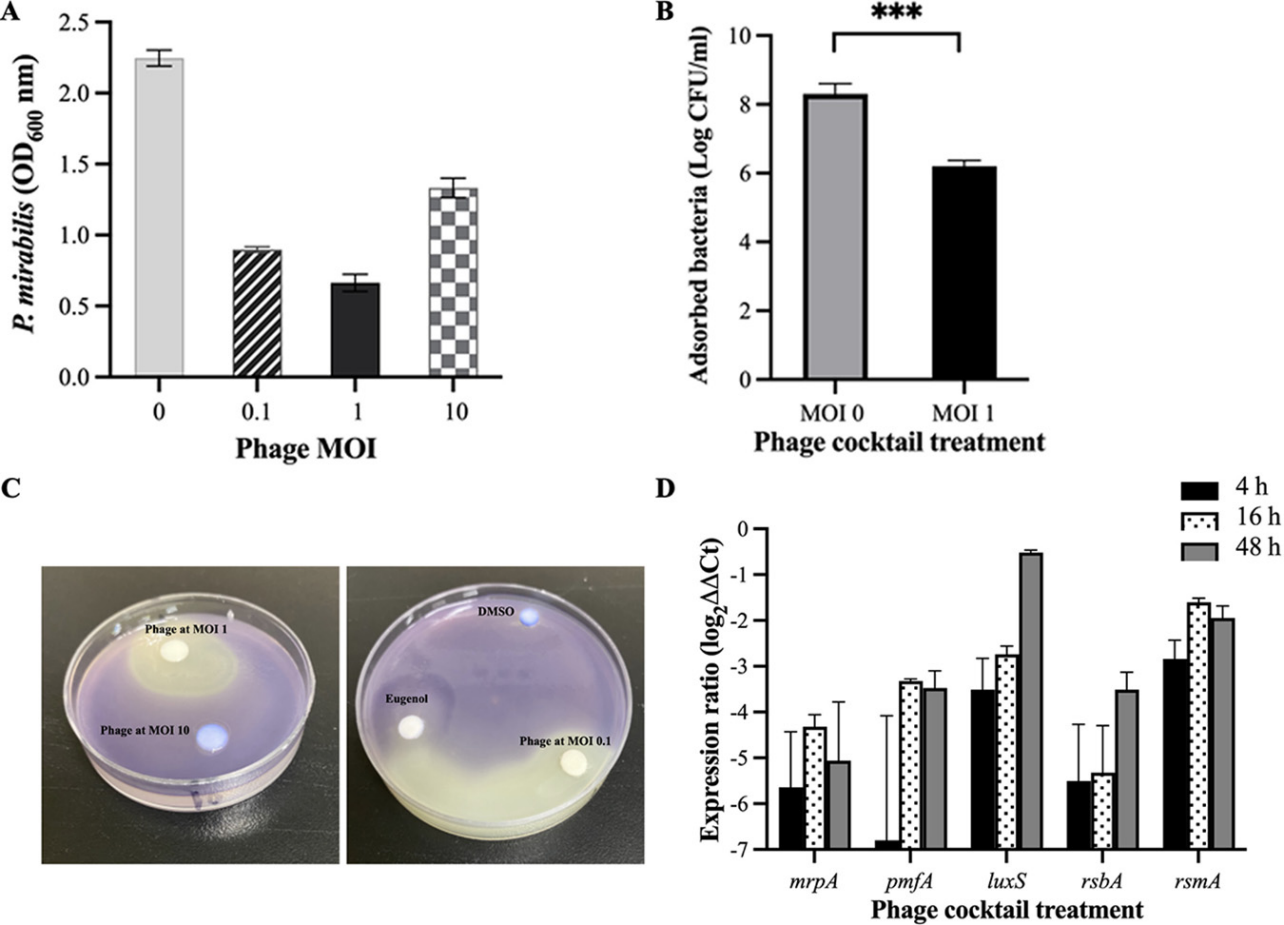
(A) Antibiofilm activity of the phage cocktail on the biomass of P. mirabilis 24 h after incubation at different MOIs. (B) Adhesion of P. mirabilis to a Vero cell line treated with phage cocktail at an MOI of 1. (C) Agar diffusion assay of phage cocktail (1:1 each phage) at MOIs of 0.1, 1, and 10 using J. lividum ATCC 12472, depicting anti-QS activity. The phage cocktail showed quorum sensing inhibition activity (indicated by outer nonpigmented ring) at an MOI of 1. Eugenol was used as a positive anti-QS control, and DMSO was used as a negative control. (D) Relative fold expression of genes related to adhesion and quorum sensing in P. mirabilis after 4, 16, and 48 h of treatment with phage cocktail using quantitative real-time PCR. The threshold cycle (*C_T_*) values of the examined genes were normalized to the 16srRNA gene. A significant reduction was demonstrated. Bars represent the SD of the treated biofilm. All experiments were performed for three biological and technical replicates, and the results are shown as the means ± SD for three independent experiments.

Initial screening of 40 P. mirabilis clinical isolates was performed to select strong biofilm-forming isolates according to the classification reported by O’Toole et al. (16). In total, 12 isolates (30%) formed a strong biofilm in 24 h. Twenty (50%) isolates were classified as moderate biofilm formers, and 8 (20%) were defined as weak biofilm-forming isolates. The strong biofilm strains (12 clinical strains) with P. mirabilis ATCC 7002 were chosen to test the phage cocktail efficacy against biofilms. After 24 h of biofilm treatment with the phage cocktail at different MOIs, we observed a significant decrease in biofilm mass compared to that of the untreated control (*P* < 0.05). The results indicate that although other MOIs could reduce P. mirabilis biofilms, an MOI of 1 has the most effective biofilm reduction. The test was repeated for 10 clinical isolates producing a strong biofilm, and consistently, a 65% reduction in biofilms was observed (Fig. 3A).

We examined the effect of phage cocktail treatment on bacterial adhesion (Fig. 3B). The results demonstrated that the phage had a 25% reduction in the adhesion of bacteria to the Vero cells.

To study the effect of phage cocktail on bacterial quorum sensing, a qualitative agar QS inhibition assay was performed. The results showed that the phage cocktail efficiently inhibited bacterial quorum sensing at an MOI of 1 (Fig. 3C). We further quantified the QS-inhibitory effect of the phage and observed that violacein production was inhibited in a concentration-dependent manner (Fig. S9). This experiment was performed at different time points (18, 48, and 72 h). In agreement with the qualitative assay, the phage cocktail at an MOI of 1 showed the highest anti-QS activity.

We used a quantitative real-time PCR (qRT-PCR) assay to examine the effect of the phage cocktail at the optimal titer of 10^8^ PFU/mL on the adhesion and quorum sensing at the gene expression level. Results showed that all of the studied genes were downregulated significantly following treatment with the phage cocktail (*P* < 0.05) (Fig. 3D).

### Phantom bladder model.

To precisely evaluate the impact of phage treatment on crystalline biofilm formation, a bladder model was used. The infection in the model was stopped after 18 h, and calcium levels on the catheter section were quantified. The findings demonstrated that the phage cocktail significantly reduced levels of encrustation (*P* < 0.001) (Fig. S10). The urine pH was measured after treatment with phage and showed a decrease after treatment.

## DISCUSSION

CAUTIs are the most serious complications of P. mirabilis infections (17). Up to 30% of all urinary tract stones (struvite) are produced by this opportunistic pathogen (18). P. mirabilis produces crystalline biofilms via the urinary tract that intermittently block the flow through catheters (19). Resistant strains have emerged in recent years because of P. mirabilis bearing antibiotic resistance elements (20). Moreover, the resistance of bacterial biofilms to antimicrobial agents and difficulties in treating biofilms compelled the search for alternative therapies. Therefore, the investigation of new methods and strategies to control MDR pathogens has been of interest. The combined use of a phage cocktail is proposed to be a more effective method for biofilm eradication since the possibility of isolating resistant strains is decreased compared to the case with phage single therapies (21).

Two lytic phages specific for P. mirabilis*-*associated CAUTIs were isolated from raw effluents of a wastewater treatment plant. The phages belong to the class *Caudoviricetes* based on the TEM morphology analysis, showing siphovirus (Isf-Pm1) and myovirus (Isf-Pm2) morphologies. These two phages showed different replication characteristics, leading to a higher burst size for Isf-Pm2. Although Isf-Pm2 showed similar to myovirus morphology, its genome-based characteristics from those deposited in GenBank suggest that it belongs to the *Straboviridae* family. Genome analysis of the isolated phages revealed that neither phage carried lysogeny-associated genes or known toxins. Taxonomic classification showed that Isf-Pm1 belongs to the pPM-01 species, which is a yet-unclassified phage of the proposed “*Pimunavirus*” genus, while Isf-Pm2 is an isolate from the same species as D5505, belonging to the *Tequatrovirus* genus.

Several phages have been utilized to combat biofilms caused by CAUTIs. For example, studies have shown that some lytic phages reduced biofilm formation in an *in vitro* catheter model system (21). It was indicated that phage cocktails could reduce biofilm formation more efficiently and for longer periods compared to phage monotherapy (22, 23). Other authors have used the phage cocktails to prevent P. mirabilis and E. coli biofilms and observed a 1-log reduction in the P. mirabilis population (22). In the case of our phage cocktail, a 4-log reduction was observed, which was higher than in other studies (22, 23). The phages’ efficacy might be related to specific phage properties. The phage cocktail developed in this study exhibited a high burst size and endolysin activity, based on burst size and endolysin assay. This protein with lytic activity was also released into the cell lysate and showed a clear pattern on the bacterial overlay which might intensify biofilm reduction (24) (Fig. S8B). The efficacy of purified phage-encoded peptidoglycan hydrolase (lysin) for the treatment of bacterial infections has also been reported (24). The polysaccharide depolymerase (PD) exhibited by tailed bacteriophages degrades polymeric components of the bacterial surface (25). These enzymes, expressed on the surfaces of phage capsids or produced by host cells during phage replication, are believed to facilitate phage attacks on biofilm communities by enabling phage penetration of the exopolymeric matrix (26). Amino acid analysis of the isolated phages revealed that both phages have putative tail spike protein, which is believed to act as a PD.

Environmental factors can affect phage stability and treatment effectiveness in phage therapy (27). The phage stability at different pHs and temperatures and against chloroform suggested application of the phage cocktail as a candidate to control CAUTIs with biological agents.

We showed that a phage cocktail at an MOI of 1 could decrease the concentration of P. mirabilis from 10^9^ CFU/mL to 10^1.6^ CFU/mL in 30 min. However, after 2 h, the number of bacteria slightly increased, which might be in terms of the emergence of phage-resistant bacteria. During phage therapy, the emergence of bacterial resistance to the phages was reported (28). We observed that lower MOIs (0.1 and 0.01) of the phage cocktail decreased the biofilm biomass to the same point in 1.5 to 2 h. Moreover, a high MOI (i.e., 10) of the phage cocktail showed the least effect to decrease the number of bacteria among the MOIs. The selection of phage-resistant bacteria was more effective under conditions when one bacterial cell was infected by several virions rather than under conditions of rare infection events in the cell population (29). Another possibility is that a high-titer phage preparation would sometimes induce the host immune system and limit phage therapy (30). In phage therapy, it is essential to use an ideal phage titer and to maximize the interaction between hosts and phage. To increase the efficiency of phage therapy, the synergistic interactions of this phage cocktail in combination with other additional therapeutic agents, such as antibiotics or antimicrobial agents, should be investigated.

Previous evaluations of phage therapy for CAUTIs have been primarily designed to evaluate the ability of phage to reduce biofilm formation in general rather than prevent catheter blockage specifically. In this case, our study is centered on blockage as a particular therapeutic endpoint and the assessment of phage utilizing a phantom bladder model. This model gives an excellent representation of the catheterized urinary system and the evaluation of phage therapy in this setting. The bladder model experiments indicate that phage treatment significantly reduced levels of calcium deposited in the catheter as crystalline biofilm formation in models of established infection. Nzakizwanayo et al. used a phantom model and showed complete eradication of P. mirabilis using a high MOI (i.e., 10) of the phage (31). The bladder model system provides an exceptionally robust evaluation of interventions to prevent blockage and encrustation. One of the main limitations of phage therapy is the rapid emergence and proliferation of bacteriophage-insensitive mutants (BIMs) (32, 33). In our study, we did not observe any regrowth of the bacterial host even 8 h after the treatment. This might reflect the application of a phage cocktail and the difference in the phage characteristics, including burst size and lag phase. Several studies have shown that phage resistance may diminish the fitness or virulence of these bacterial variants and therefore facilitate clearance by the immune system (28). Applying cocktails composed of phages that target different cell receptors has been suggested to improve phage therapy by extending the host range and reducing resistance (34). This is particularly important in biofilms, in which applying the cocktails rather than a single phage can delay (32) or even prevent (35) the emergence of resistant bacterial variants.

The expression levels of selected genes involved in the biofilm formation and QS in phage-treated biofilms were evaluated by qRT-PCR. The data revealed that the phage cocktail significantly affected the downregulation of adhesion-associated genes that contributed to biofilm formation. Furthermore, in line with the results obtained in violacein inhibition assays, the observed downregulation of *luxS* could be explained by bacterial elimination and, consequently, the reduction in receptors. The bacterial adhesion to Vero cells was examined after phage treatment compared to that of untreated bacteria and showed a significant reduction. This result confirmed the data obtained by the qPCR. Other studies evaluated the effect of mutation of the *mrpA* gene on the adhesion of P. mirabilis in two cell lines and observed a significant reduction in the adhesion of P. mirabilis to the cells, which is in line with our results (36). According to these results, phage cocktails might be helpful as therapeutic alternatives to antibiotics to combat P. mirabilis-associated CAUTIs. However, more investigation, including *in vivo* assay and other analyses, is needed to confirm the effectiveness of phages. This combined approach will control the development of bacterial resistance during treatment.

In conclusion, the specificity of the isolated phages, the absence of genes associated with lysogeny, and the efficient antibiofilm reduction obtained using the *in vitro* bladder model support using both phages Isf-Pm1 and Isf-Pm2 to control P. mirabilis biofilm formation on silicone catheters.

## MATERIALS AND METHODS

### Isolation of bacteria from the catheter.

In this study, 385 nonduplicate catheters (7 days to 15 days) were collected from patients admitted to the reference intensive care units (ICUs) in hospitals of Isfahan, Iran. The catheters were collected from patients who did not have primary urinary tract infections (UTIs) at the time of admission, and the minimum catheterization time was 7 days. P. mirabilis was isolated from the catheter biofilm according to a previously described method (37, 38). All isolates were cultured on tryptic soy broth (TSB) and MacConkey agar, followed by incubation at 37°C. The identification of the bacterial isolates was confirmed by conventional biochemical methods, followed by 16S rRNA and *ureG* gene sequencing (38). The antimicrobial susceptibility testing of clinical isolates was performed according to guidelines provided by the Clinical and Laboratory Standards Institute (CLSI) (39).

### Isolation of bacteriophage.

Municipal wastewater samples were collected from a sewage treatment plant as described previously (40). Briefly, 10 mL of wastewater sample was filtered through a 0.22-μm-pore-size membrane filter (Millipore, Göttingen, Germany) and mixed with an equal volume of 2× nutrient broth (NB; Merck, Germany) supplemented with 1 mM MgSO_4_ and 1 mM CaCl_2_ (Merck, Germany) containing 1 mL of an overnight culture (10^8^ CFU/mL) of P. mirabilis ATCC 7002 and incubated at 37°C for 18 to 20 h, with shaking (150 rpm). The culture was centrifuged at 10,000 × *g* for 20 min at 4°C to remove cell debris and filtered through a syringe sterile filter (0.22 μm). The isolated phages were plaque purified, and titers were determined by a double-layer agar method (41). In brief, 200 μL of the filtrate was mixed with 2.5 mL of soft nutrient agar (0.7% agar) containing 100 μL of P. mirabilis (10^8^ CFU/mL). The mixture was overlaid onto solidified nutrient agar (1.5% agar) and incubated for 24 h at 37°C. A single plaque was picked from the soft top agar by sterile Pasteur pipette and soaked in 1 mL of SM buffer (50 mM Tris-Cl, 100 mM NaCl, 8 mM MgSO_4_ [pH 7.5]). Several rounds of double-layer plaque assay were performed until single-plaque morphology was observed (42). The phage titer was determined by the standard double-layer agar method, and the titer was reported as PFU per milliliter (43).

### Transmission electron microscopy (TEM).

The high-titer phage lysates (1.5 mL) were concentrated with polyethylene glycol (PEG)-NaCl (20% PEG 8000, 2.5 M NaCl), and 10 μL of the concentrated phage suspension was dropped on a carbon-coated copper grid and negatively stained with 2% uranyl acetate. Phages were examined under a JEM-1400 Flash transmission electron microscope (JEOL, USA) at a voltage of 80 kV.

### Phage adsorption rate.

The adsorption rate and adsorption rate constant (*k*) of the isolated phages were determined by the adsorption curve according to the method of Hyman and Abedon (44). Briefly, exponentially growing P. mirabilis ATCC 7002 was infected with the phage at a multiplicity of infection (MOI) of 0.1 and incubated at 37°C. Samples were collected every minute postincubation for 10 min, followed by centrifugation at 10,000 × *g* for 5 min at 4°C. The supernatant was filtered through a 0.22-μm filter, and the unabsorbed phages in the supernatant were titrated by the double-layer agar plaque assay for plaque counting. The experiments were undertaken independently in triplicate.

### One-step growth experiment.

To determine the latent period and the burst size of the isolated phages, a one-step growth experiment was performed (45). One milliliter of P. mirabilis (10^8^ CFU/mL) in exponential phase was mixed with the phage at an MOI of 0.01 and incubated for 10 min. Unabsorbed phages were removed by brief centrifugation (6,000 × *g* for 10 min), and 50 μL of the pellet was transferred to 50 mL of NB and incubated at 37°C. Samples were collected at 10-min intervals for an overall time of 120 min. Supernatants were filtered and diluted, and then the number of phages was immediately determined using the double-layer agar method (41). Burst size was calculated as the ratio of phage titer to the number of initial infected bacterial cells. This experiment was carried out three times with a triplicate plaque assay.

### Determination of phage stability.

For the pH stability test, 10^8^ PFU/mL of the phage was incubated at 37°C for 1 h in SM buffer at different pH values (3, 5, 7.9, 11, and 13). Next, the phage titer was determined using the double-layer agar method (41). For thermal stability, phage aliquots were collected at 5-min, 15-min, 45-min, and 1-h intervals at various temperatures (4, 25, 37, 50, and 70°C). To test chloroform stability, 1 mL (1 × 10^8^ PFU) of the phage was mixed with 0.4 mL of chloroform, and the phage was collected and titrated after a 1-h incubation at room temperature.

### Determination of optimal phage titer.

A 10-fold serial dilution series of the phage cocktail (10^6^ to 10^9^ PFU/mL), equal to MOIs of 0.001 to 10, was prepared and inoculated to an equal volume of freshly prepared bacteria (10^8^ CFU/mL) separately. The mixture was incubated at 37°C with shaking (150 rpm). P. mirabilis incubated with NB without the phage was used as a control sample. One milliliter of the sample was taken every 30 min for 90 min and centrifuged at 12,000 × *g* for 5 min. Then, the pellet was washed with phosphate-buffered saline (PBS) and resuspended in 1 mL of NB. The bacterial suspension was serially diluted and spread on nutrient agar (1.5%). The bacterial colonies were counted on the plate and represented as CFU per milliliter, as previously described by Tang et al. (46).

### Bacterial reduction assay.

A 1-mL culture of P. mirabilis ATCC 7002 with an optical density at 60 nm (OD_600_) of 0.1 (10^8^ CFU/mL) was infected with the phage cocktail at an MOI of 1 in a flask containing 100 mL of NB and incubated at 37°C with shaking at 150 rpm. As a control, P. mirabilis was incubated with NB without the phage cocktail. The bacterial growth was monitored by recording OD_600_ every 20 min for 8 h, as previously described (47).

### Phage whole-genome sequencing.

Bacteriophage DNA was extracted using the phenol-chloroform method (48) and subsequently sequenced using Illumina MiniSeq as described by Makalatia et al. (49). Genome assembly was performed using SPAdes (Galaxy v3.12.0+galaxy1), followed by alignment of the phage genomes to their most closely similar phages as identified by BLASTn (accessed in October 2021). Finally, the phage genome was annotated using RASTtk (PATRIC v3.6.12) and manually curated by BLASTp (accessed in November 2021). The final GenBank files were submitted to the NCBI database (accession numbers OL741431 and OL741432). We manually screened the annotated proteins for lysogeny-related proteins and verified the lifestyle of the phage using the Phage.AI platform (50).

### SDS-PAGE analysis.

SDS-PAGE was used to visualize the structural proteins of the isolated phages. The standard protocols for SDS-PAGE analysis were adapted from Laemmli’s gel method (51). The phage particles were filtered through a 0.22-μm filter and concentrated by precipitation with PEG and ultracentrifugal filtration (Amicon; Millipore Sigma-Aldrich, USA) at 13,000 × *g* and 4°C for 45 min, and the concentrated phage lysate was boiled with sample loading buffer. SDS-PAGE was carried out on a 12% gel with staining with Coomassie brilliant blue (Ameresco, USA).

### Lysis protein analysis under nondenaturing conditions.

The concentrated phage lysate (as described above) was mixed with protein loading buffer without β-mercaptoethanol. The samples were subjected to PAGE without boiling. The resolved gel was placed onto an agar-coated plate, in which soft agar mixed with the P. mirabilis had been previously poured onto the gel and incubated at 37°C overnight. Clear zones on the overlay indicate lytic proteins (52).

### Cell survival assay.

We investigated the toxicity of the phage cocktail in Vero cells. Vero cells (2.5 × 10^4^ well/plate) were plated in a 24-well plate in the presence of 100 μL of Dulbecco’s modified Eagle’s medium (DMEM; Gibco, USA) supplemented with 5% fetal calf serum (FCS; Gibco, USA) at 37°C in 5% CO_2_ (15). When cells reached 70 to 80% confluence, 10^8^ CFU of P. mirabilis ATCC 7002 was added to each well, followed by the addition of the phage at different MOIs (0.01, 0.1, and 1). We used two controls: Vero cells treated with 10^8^ PFU of the phage in the absence of P. mirabilis and Vero cells treated with P. mirabilis without addition of the phage. In another experiment, the cells were first infected with 10^8^ CFU of P. mirabilis. After 2 h postincubation, the phage was added to the infected cells, and the number of living cells was counted (53).

### Biofilm reduction assay by phage cocktail.

P. mirabilis ATCC 7002 and 10 biofilm-forming P. mirabilis clinical isolates were used to examine the efficacy of the phage cocktail to reduce established biofilms. A bacterial suspension (1 × 10^8^ CFU/mL) of P. mirabilis was prepared in NB. Then, 100 μL of the suspension was added to a 96-well flat-bottom polystyrene plate and incubated at 37°C for 72 h to facilitate biofilm formation. The planktonic bacteria were subsequently removed, and a phage cocktail containing equal concentrations of Isf-Pm1 and Isf-Pm2 (total final concentrations, 10^8^, 10^7^, and 10^6^ PFU/mL) was added to each well in triplicate. As a control sample, NB was added to the wells without the phage. The plate was incubated at 37°C for 24 h. The biofilm mass was assessed by crystal violet staining (54).

### Adhesion assay.

Semiconfluent Vero cells, seeded in 24-well plates, were treated or not with the phage cocktail (10^8^ PFU/mL) and infected with 40 μL of P. mirabilis ATCC 7002 (10^8^ CFU/mL) and 960 μL of DMEM. After incubation at 37°C under 5% CO_2_ for 2 h, the wells were washed with PBS three times to remove nonadherent bacteria. Then, the cells in each well were treated with 500 μL of 0.025% Triton X-100 solution (Sigma-Aldrich) for 5 min at 37°C in 5% CO_2_ to detach and lyse the cell monolayer. The cell lysates were homogenized by adding 500 μL of PBS and pipetting. Serial dilutions were prepared and plated on the agar. The colonies of bacteria were counted after overnight incubation of the plates. The effect of the phage cocktail was determined by comparing colony counts obtained from lysates of cell cultures with and without phage and reported as CFU per milliliter (36). All samples were tested in triplicate, and each experiment was performed three times.

### Qualitative agar QS inhibition assay.

The quorum sensing (QS) inhibitory activity of the phage cocktail was investigated using the Janthinobacterium lividum biosensor system. In the wild-type strain J. lividum ATCC 12472, the production of a purple pigment, violacein, is under the control of a QS system. Five milliliters of molten soft LB agar (0.3% [wt/vol]) was inoculated with 50 μL of J. lividum ATCC 12472 grown overnight in Luria-Bertani (LB) broth. The agar-culture solution was immediately poured over the surfaces of prewarmed LB agar plates. Then, 20 μL (10^6^, 10^7^, and 10^8^ PFU/mL) of phage cocktail was pipetted onto sterile paper discs, dried, and placed on the solidified agar. Plates were incubated overnight at 25°C and examined for violacein pigment production. QS inhibition was detected by a colorless, opaque, but viable halo around the discs (loss of pigmentation). Dimethyl sulfoxide (DMSO) was used as a control (55).

### Quantitative anti-QS activity—violacein inhibition.

J. lividum was cultured aerobically in LB at 25°C with or without the addition of increasing concentrations of phage (MOIs of 0.1, 1, and 10). Eugenol (6.25 mg/mL; Sigma, St. Louis, MO, USA) was used as a standard positive control for the QS inhibitor. One milliliter of the overnight culture of J. lividum ATCC 12472 was centrifuged (12,000 × *g*, 10 min) to precipitate the insoluble violacein. The culture supernatant was discarded, and the pellet was evenly resuspended in 1 mL of DMSO. The solution was centrifuged (12,000 × *g*, 10 min) to remove the cells, and the violacein was quantified at OD_585_ using a UV-visible (UV-Vis) spectrophotometer (UV-1800; Shimadzu, Kyoto, Japan). The percent violacein inhibition was calculated by the following formula (55):

$$\%\text{ violacein inhibition}=\left({\text{control OD}}_{585}-{\text{test OD}}_{585}/{\text{control OD}}_{585}\right)\times \text{1}00$$

### Efficacy of phage cocktails in a bladder phantom model.

*In vitro* bladder models, initially described by Stickler et al., were set up and operated as described previously (18). The model consisted of a double-walled glass chamber (the bladder) maintained at 37°C by a water jacket supplied from a circulating water bath. The model was sterilized by autoclaving, and then catheters (size 18 all-silicone Foley catheters) were inserted into the bladder via an outlet in the base of the glass chamber before retention balloons were inflated with 10 mL of sterile water. Catheters were subsequently attached to a drainage and reservoir bag. A sterile urine sample from a healthy male volunteer with no history of UTIs was filtered (through 0.2-μm-pore-size filters) and supplied to the bladder via a peristaltic pump. In this way, a residual volume of urine (30 mL) collects in the bladder below the level of the catheter eyehole. P. mirabilis ATCC 7002 cell suspensions (10^8^ CFU/mL) were inoculated directly into the residual bladder urine, representing late-stage infection, and left for 1 h to permit cells to establish the infection within the system. At 45 min postinoculation, the test models were treated with a single dose of 2 × 10^10^ PFU of the two-phage cocktail (1 × 10^10^ PFU/mL of each phage) in a total volume of 1 mL, and flow was restored 15 min later. The overflow drains through the catheter to the collecting bag as urine is supplied to the model. The numbers of viable cells present in the residual bladder medium were enumerated at the start and end of the experiments, and the pH was checked at the beginning and end of the experiments by sampling the medium from the bladder. Then, the calcium deposition on the catheters was calculated by flame photometry 18 h posttreatment (31).

### Quantification of adhesion and QS genes by real-time PCR.

P. mirabilis ATCC 7002 was grown on an agar plate, scraped, and inoculated into 10 mL of pooled urine from a healthy male volunteer with no history of UTIs and incubated overnight. Then, 1 mL of urine-grown bacteria (OD_640_ = 0.1) was transferred to 10 mL of fresh pooled urine supplemented with phage (10^8^ PFU/mL) at 37°C for 4, 16, and 48 h. The respective control group did not include the test component. The OD_640_ of 0.1 was adjusted in pooled urine, and 1 mL of each culture was transferred to 2-mL tubes. The suspension was centrifuged (10,000 × *g*, 5 min), the supernatant was removed, and the pellet was resuspended in 500 μL of PBS. Total RNA was extracted from the bacterial cells with the RNeasy minikit (Qiagen, Germany), according to the manufacturer’s protocol. The cDNA was synthesized with a Jena Biosciences kit (Germany) according to the manufacturer’s instruction and was ready for qPCR. qPCR was performed for five genes involved in biofilm formation and adhesion (*map*, *pmfA*, *luxS*, *rsmA*, and *rsbA*) and a reference gene (16S rRNA) using the RealQ Plus 2× master mix green Ampliqon (Denmark) according to the manufacturer’s instructions in a real-time PCR instrument (Applied Biosystems StepOne and StepOnePlus real-time PCR). The expression of selected genes was calculated relative to the calibrator (P. mirabilis ATCC 7002 that had been cultured for 4 h) and normalized to the expression of the reference gene (16S rRNA for each organism). The primer sets used in this study are summarized in Table 2. The expression fold change of a target gene = 2ˆΔΔ*Cq* (where Δ*Cq* = *Cq* [target gene] − *Cq* [reference gene], and ΔΔ*Cq* = Δ*Cq* [test] − Δ*Cq* [calibrator]; *Cq* is quantification cycle).

**TABLE 2:** Sequences of primers used in this study

Gene	Primer sequence (5′–3′)^*a*^	Product (bp)	Reference
*mrpA*	F, TGCTGCATTAAAAGATGGTGGC; R, TTTGTTTACCACCCGCATCG	200	This study
*pmfA*	F, GGCTGCGGCTTTAGTATTTG; R, GGCTTGAAGATGCTGCTAATC	146	This study
*luxS*	F, AAAGCCATGCCTGAGAAAGG; R, CGACATCCCATTGGCGAAATA	116	This study
*rsmA*	F, AGCCTTTAATCAGCGCCGTA; R, GCGTGTTGCTGTTGTGATGA	165	This study
*rsbA*	F, CGCTATCACGCTAACCAACTA; R, GCGTCCTTCAAGCCAATAAAC	120	This study
16S rRNA	F, ATGTTGGGTTAAGTCCCG; R, CTAGCGATTCCRRCTTCA	256	56

a F, forward; R, reverse.

### Statistical analysis.

The data were analyzed by one-way and two-way analysis of variance (ANOVA) with Tukey’s multiple-comparison test using GraphPad Prism version 9.3.1 for Windows (GraphPad Software, Inc., La Jolla, CA, USA). A *P* value of ≤0.05 was considered significant.

### Data availability.

The genome sequences of Isf-Pm1 and Isf-Pm2 phages were deposited in the GenBank database under accession numbers OL741431 and OL741432, respectively. Source data are provided with this paper. We declare that all other data supporting the findings of this study are available within the article and supplemental data files at https://www.researchgate.net/publication/363250612_Supplementary_Figures_and-T00les.
